# Sex‐specific associations of *Roseburia* with uric acid metabolism and hyperuricemia risk in females

**DOI:** 10.1002/imo2.70016

**Published:** 2025-04-01

**Authors:** Zhihan Yang, Zeng Zhang, Mohan Chen, Jin Hu, Yingqi Qiu, Shijia Lin, Xiaolu Zhou, Jiachao Zhang, Shuaiming Jiang

**Affiliations:** ^1^ School of Food Science and Engineering, Key Laboratory of Food Nutrition and Functional Food of Hainan Province Hainan University Haikou Hainan China; ^2^ Bo'ao Yiling Life Care Center Qionghai Hainan China; ^3^ Haikou People's Hospital Haikou Hainan China; ^4^ One Health Institute Hainan University Haikou Hainan China

**Keywords:** females, gender disparity, gut microbiota, hyperuricemia, population cohort, roseburia

## Abstract

Hyperuricemia (HUA) affects approximately 14% of the population, with a marked gender disparity showing lower incidence in females. While the role of gut microbiota in HUA has been extensively studied in males, its influence in females remains unclear. This study recruited 623 participants, including 270 females, to explore this gap using 16S rDNA sequencing. We found that *Roseburia* was significantly enriched in healthy females and negatively correlated with estrogen (E2) and uric acid (UA) levels. Validation using three published cohorts (256 samples) confirmed *Roseburia* as a critical genus for UA regulation. Mechanistically, *Roseburia* was positively correlated with the xanthine‐glycine metabolic pathway, which may influence UA production in females. These findings shed light on gender differences in HUA incidence from a gut microbiota perspective and propose *Roseburia* as a promising target for probiotic‐based prevention of HUA in females.

## Highlights

- Analysis of 623 population samples suggests microbiological factors behind lower hyperuricemia (HUA) incidence in females.
- *Roseburia* is a sex‐specific genus that may benefit the mitigation of HUA in females.
- *Roseburia* is positively correlated with E2 levels and negatively correlated with uric acid (UA) levels.
- The xanthine‐glycine pathway is positively correlated with the abundance of *Roseburia*, which might contribute to lower UA levels through this pathway.

## Graphical abstract

Researchers recruited 623 participants, including 270 females, and used 16S rDNA sequencing to investigate gender differences in hyperuricemia. In healthy females, *Roseburia* was enriched, positively correlating with estrogen and negatively with uric acid, suggesting it is a potential biomarker affecting uric acid levels in females.

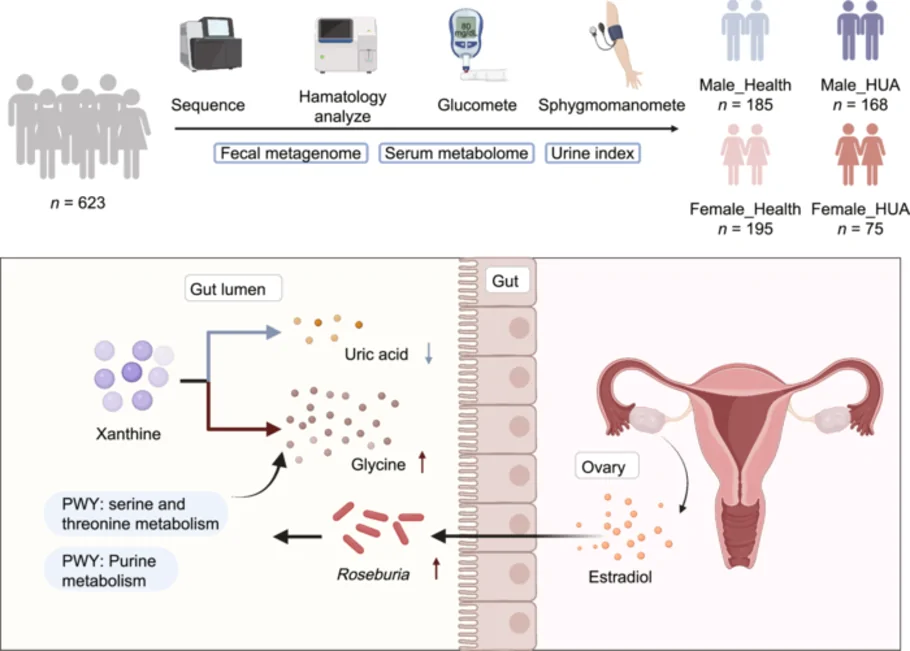

## 1 INTRODUCTION

Hyperuricemia (HUA), a metabolic disorder marked by elevated levels of serum uric acid (UA), was a known precursor to gout, chronic kidney disease, and cardiovascular disease [1]. HUA is a serum UA concentration >420 μmol/L in males and >360 μmol/L in females [2]. HUA could arise from disruptions in purine metabolism [3], impaired excretion, or overproduction of UA [4]. Notably, although HUA can affect both sexes, the incidence rate is significantly higher in males compared to females, at 24.4% and 3.6%, respectively [2], indicating a clear gender disparity in HUA prevalence. In recent years, with the rapid rise in HUA incidence globally and the trend towards younger onset ages, the need to understand these gender differences has become particularly urgent [5]. This understanding is crucial for better prevention and treatment strategies tailored to different genders, improving patient outcomes.

Approximately 70% of the body's UA was excreted through the kidneys, while the remainder was primarily excreted via fecal or further metabolized by the gut microbiota [6]. The gut microbiota and its metabolites lowered serum UA levels by influencing purine and UA catabolism, regulating intestinal UA transporters and excretion, and maintaining intestinal barrier permeability [7]. The gut microbiota not only promotes the breakdown and excretion of UA by participating in purine metabolism but also helps maintain UA balance by regulating the expression of UA‐related genes and through the modulating effects of probiotics and prebiotics [8, 9]. These mechanisms influence UA production and excretion pathways, offering new potential targets and strategies for treating HUA. Several studies demonstrated that the gut microbiota played a significant role in the development and progression of HUA [7, 10, 11, 12]. *Lactobacillus rhamnosus* was reported to reduce UA by decreasing the activity of xanthine oxidase in the liver [13], and *Lactobacillus fermentum* directly degraded UA to alleviate HUA [14].

Research has shown that males and females differ in their gut microbiota composition and exhibit distinct biomarkers in various metabolic diseases, suggesting a gender‐dependent influence on disease development [15]. As such, gender differences in gut microbiota may have been crucial in the sex‐specific variations observed in human diseases. Sex hormones were known to affect gut microbiota composition, and similar gut microbiota could function differently between the sexes [16]. While HUA affects both sexes, its prevalence is notably higher in males (24.4%) compared to females (3.6%) [2]. Most existing studies have predominantly focused on male populations. For instance, in a study by Sheng et al. [17], which included 187 male participants, significant alterations in the structure and function of the gut microbiota were observed in males with HUA. Similarly, Shao et al. [18] collected samples from 52 males and identified altered metabolic signatures related to UA excretion, purine metabolism, and inflammatory responses through metagenomic and metabolomic analyses. However, research on the role of gut microbiota in female patients with HUA is still insufficient. Therefore, further studies are needed to explore the differential impacts of gut microbiota on HUA in male and female patients. In this study, 623 subjects were recruited, including 270 females. Their fecal, serum, and urine samples were collected, and the sex‐specific role of gut microbiota in HUA was analyzed using 16S rDNA sequencing. This study explores the sex differences in gut microbiota composition and its potential association with HUA.

## 2 RESULTS

### Sex differences in the effects of HUA on hosts

**FIGURE 1:**
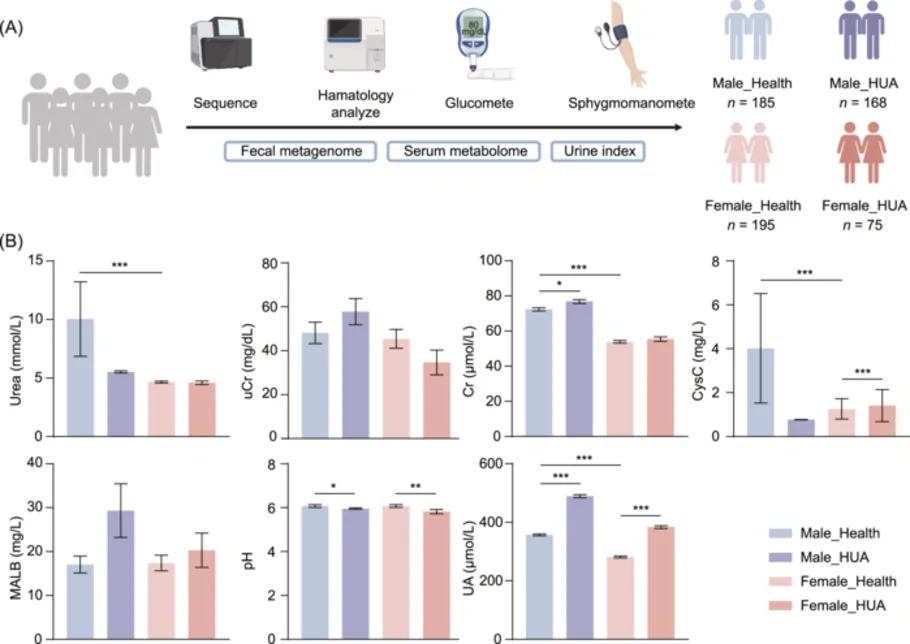
Flowchart of the clinical trial. (A) Experimental design: 623 individuals were collected from Hainan, China, with all female participants being premenopausal. Fecal and serum were collected from the subjects. The gut microbiota was sequenced using 16S rDNA, and differential metabolites in the serum were identified by LC‐MS. Data from Clinical tests were also collected from the participants. Based on the UA levels, the samples were categorized as Male_Health (healthy males, *n* = 185), Male_HUA (males with HUA, *n* = 168), Female_Health (healthy females, *n* = 195), and Female_HUA (females with HUA, *n* = 75). (B) Levels of renal (urea; Cr: creatinine; CysC: cystatin C; UA: uric‐acid) and urinary (uCr: urinary creatinine; pH; MALB: microalbumin) indicators in four groups of subjects. Statistical significance is denoted by *p < 0.05, **p < 0.01, ***p < 0.001, analyzed by the Mann–Whitney *U* test. HUA, hyperuricemia.

In this study, a total of 623 subjects were recruited and categorized into four groups based on UA levels: Male_Health (healthy males, *n* = 185), Male_HUA (males with HUA, *n* = 168), Female_Health (healthy females, *n* = 195), and Female_HUA (females with HUA, *n* = 75). To assess the health status and gut microbiota of the participants, clinical indicators related to liver and kidney function, lipid levels, and hormone levels were collected, and 16S rDNA sequencing was performed on stool samples to gain insight into the composition and metabolic capacity of the gut microbiota (Figure 1A). In particular, levels of urea, Creatinine (Cr), cystatin C (CysC), and UA were significantly higher in Male_Health than in Female_Health (*p* < 0.001) (Figure 1B). Liver‐related indices, such as alkaline phosphatase (ALP) and cardiovascular indices, including low‐density lipoprotein cholesterol (LDL‐C), apolipoprotein B (ApoB), and cholesterol (CHOL), showed significant differences between Male_HUA and Male_Health, but no differences were found in females (Table S1). Among them, the indicators related to cardiovascular diseases are higher. Between Female_HUA and Female_Health, changes were observed in immune‐related immature granulocyte (IG), cardiovascular‐related high‐density lipoprotein cholesterol (HDL‐C), and apolipoprotein A1 (ApoA1), and hematologic parameters such as red blood cell (RBC), hematocrit (HCT), and hemoglobin (Hb), none of which showed significant alterations in the male group. These findings suggested that specific clinical indicators, including liver function, may exhibit sex‐specific differences in HUA patients.

### Gut microbiota was significantly associated with sex‐specific levels of HUA

**FIGURE 2:**
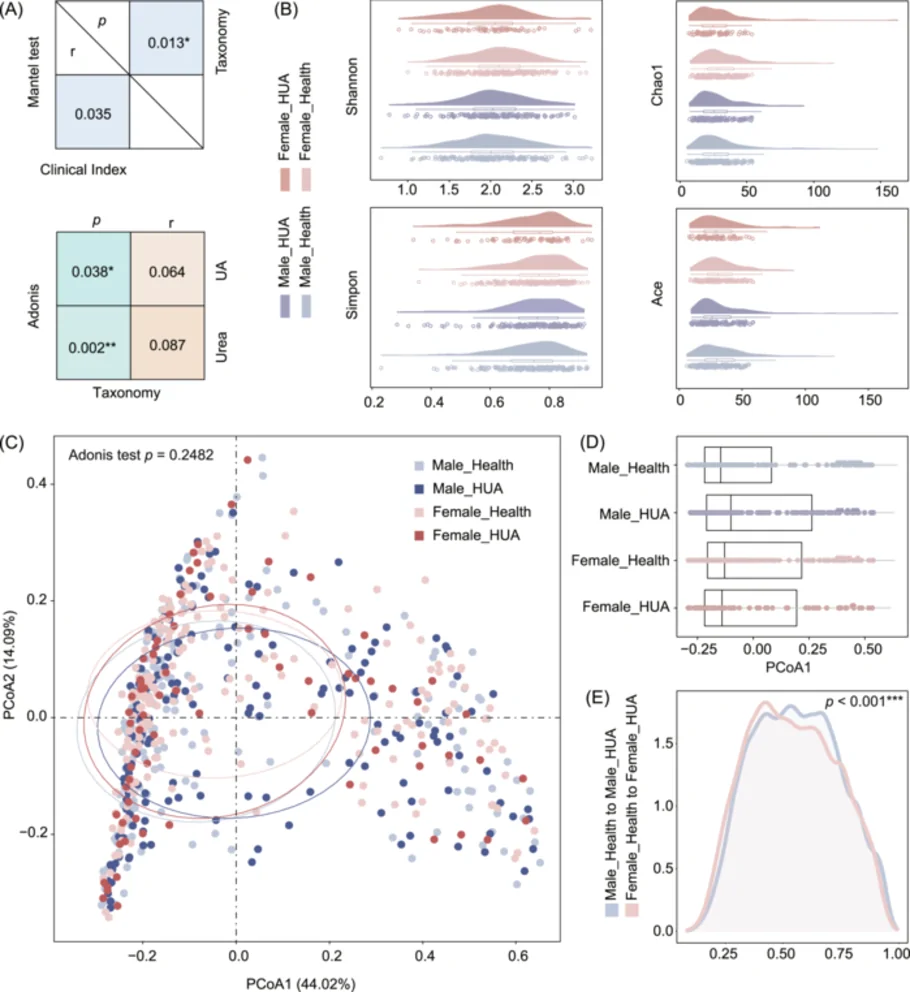
Differences in the structure of gut microbiota. (A) Analysis of the effect of gut microbiota on UA and urea results using the Mantal and Adonis test. (B) In subjects, the gut microbiota's alpha diversity is analyzed (Simpson, Shannon, Chao1, and ACE index). (C) PCoA showed a difference in microbial composition based on the Bray–Curtis distance. Each point represents the composition of the microbiota of one sample. (D) Comparison of PCoA1 in four groups. (E) Quantitative comparison of differences in the distance (based on the Bray–Curtis distance) between healthy and HUA gut microbiota in males and females. HUA, hyperuricemia; UA, uric acid.

The subjects' gut microbiota and clinical indicators were comprehensively analyzed, and the Mantel test results showed a significant correlation between gut microbiota and clinical indicators (*p* = 0.013) (Figure 2A). The Adonis test for individual data matrices similarly indicated that the gut microbiota was significantly correlated with UA and urea levels in the host, suggesting that gut microbiota may influence these clinical indicators and potentially serve as indicative markers of HUA (Figure 2A). Despite these correlations, alpha diversity analysis, including the Shannon, Simpson, Chao1, and ACE index, showed no significant differences in gut microbiota diversity among the four groups (Figure 2B). In contrast, the ACE and Chao1 indices, which reflect genera richness, exhibited higher values in healthy populations than in diseased populations in both male and female cohorts. Meanwhile, the Shannon and Simpson index, which indicated genera evenness and diversity, were highest in the Female_Health group (Figure 2B). Principal coordinate analysis (PCoA) based on the Bray–Curtis distance showed no significant differences in the structure of the gut microbiota between the four groups: Male_Health, Male_HUA, Female_Health, and Female_HUA groups (Adonis test, *p* = 0.2482) (Figure 2C,D). Further quantifying the Bray–Curtis distance revealed a more pronounced difference in gut microbiota structure between Male_Health and Male_HUA. In contrast, the difference between Female_Health and Female_HUA was relatively minor (*p* < 0.001) (Figure 2E). These results suggested that HUA had a more significant impact on the gut microbiota in males than in females.

### Enrichment of bacteria that produce short‐chain fatty acids (SCFAs) in the intestinal tract of Female_Health

**FIGURE 3:**
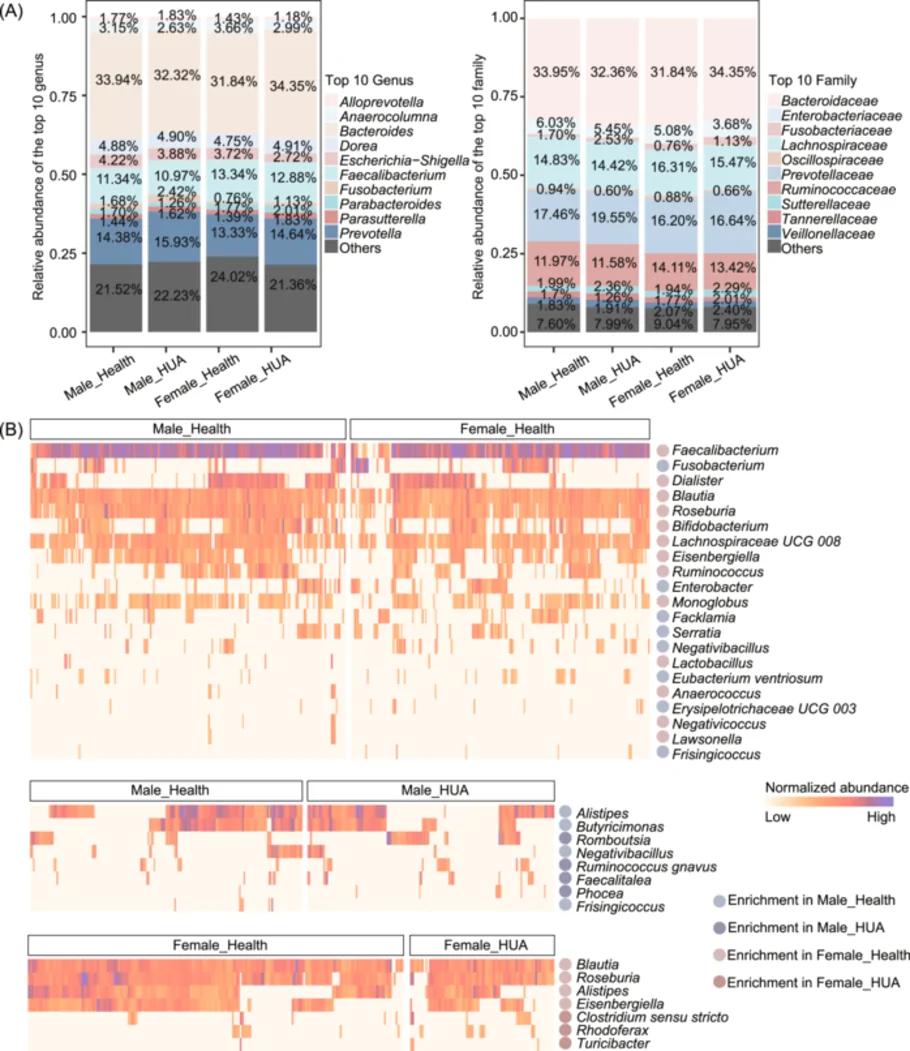
The Female's gut microbiota differed from that of the male and affected characterization in hyperuricemia (HUA). (A) The top 10 genera and family's abundance of gut microbiota in subjects. (B) Heatmaps showed differences in gut microbiota between the two groups. Grid color depth represented the relative abundance of species (purple for high abundance, yellow for low abundance). The circle colors depicted the genus enrichment across different groups: blue for enrichment in the Male_Health, purple for enrichment in Male_HUA, pink for enrichment in Female_Health, and red for enrichment in Female_HUA. *p < 0.05, **p < 0.01, ***p < 0.001, analyzed by the Mann–Whitney *U* test.

**FIGURE 4:**
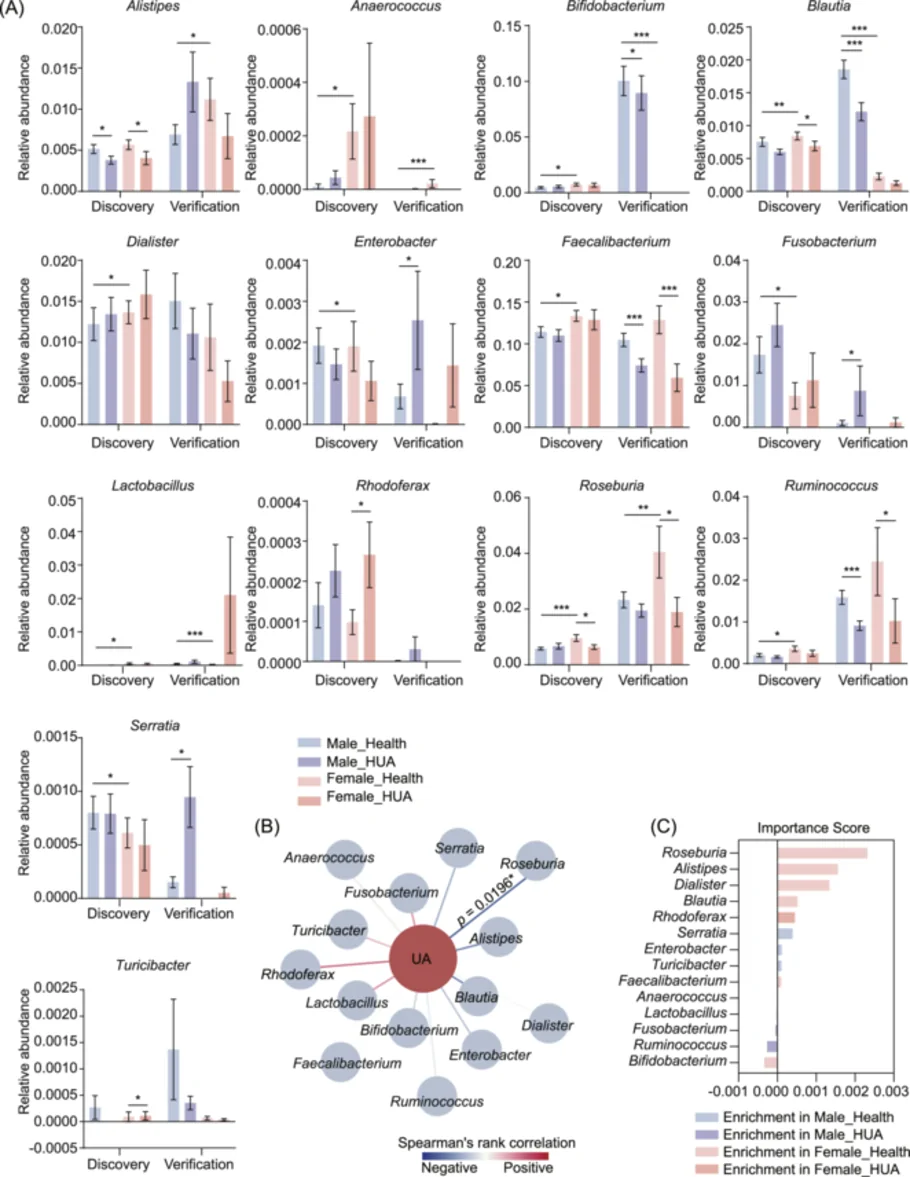
Altered and predictive biomarker of the gut microbiota in female HUA subjects. (A) Fourteen genera were annotated in the validation cohort, and they differed between Male_Health, Male_HUA, Female_Health, and Female_HUA in the discovery cohort. (B) Correlations between genera and UA. The color of the edges represented the degree of correlation, with blue indicating a negative correlation and red indicating a positive correlation. The darker the color, the stronger the correlation. (C) Random forest analysis of the contribution of 14 important genera to UA disease and healthy populations. HUA, hyperuricemia; UA, uric acid.

Based on the established significant association between gut microbiota and HUA, the relationship between gut microbiota and sex differences in HUA was explored. The top 10 genera and families in the gut microbiota of all subjects were listed (Figure 3A). For instance, *Bacteroides* and *Parabacteroides* were higher in the Male_Health group than in the Male_HUA group, whereas in the Female_HUA group, they were higher than in the Female_Health group. The relative abundance of *Alloprevotella* and *Lachnoclostridium* was higher in the Female_Health group than in the Female_HUA group, while the opposite was observed in the male group. At the family level, Bacteroidaceae and Tannerellaceae were more abundant in the Male_Health group than in the Female_HUA group (Figure 3A). Overall, the gut microbiota exhibited sex differences in both genera and families. This finding suggested that HUA influenced gut microbiota composition, and this alteration was closely linked to gender differences. To identify the gut microbiota genera with potential protective roles against HUA in females, we first compared the differences between Male_Health and Female_Health (Figure 3B), thereby identifying genera specific to each gender. The gut microbiota genera screened from healthy males and females may represent those with potential protective significance for females without disease. Compared to Male_Health, several beneficial genera were significantly enriched in the Female_Health cohort, including *Faecalibacterium*, *Dialister*, *Blautia*, *Roseburia*, and *Bifidobacterium*. Subsequently, by comparing the gut microbiota differences between Female_Health and Female_HUA, we further identified the genera affected by HUA in females, thus capturing disease‐specific microbial characteristics within the female population (Figure 3B). The results indicated that *Blautia*, *Roseburia*, *Alistipes*, and *Eisenbergiella* were enriched in the Female_Health cohort, producing SCFAs that reduce intestinal permeability and decrease pro‐inflammatory factors [19, 20, 21, 22, 23]. Among them, it had been reported that *Alistipes* and *Roseburia* were negatively correlated with UA levels [24]. To further validate our research, 256 data on gut microbiomes from three published studies on HUA populations were collected to examine the abundance differences of these genera across different populations (Figure 4A). Fourteen genera were also annotated in the validation cohort, with *Alistipes*, *Blautia*, *Roseburia*, *Faecalibacterium*, and *Ruminococcus* being enriched in Female_Health, while *Serratia*, *Rhodoferax*, and *Enterobacter* were more abundant in Male_HUA (Figure 4A).

### *Roseburia* as a key biomarker for protection against HUA in females

To further screen for key biomarkers contributing to sex differences in HUA, the correlation between these genera and UA levels was calculated (Figure 4B). *Serratia*, *Roseburia*, *Alistipes*, *Blautia*, *Dialister*, *Bifidobacterium*, *Enterobacter*, *Faecalibacterium*, and *Ruminococcus* were negatively correlated with UA levels. The negative correlation between *Roseburia* and UA levels was significant (*p* = 0.0196) (Figure 4B). Subsequently, a model of the random forest was applied to assess the predictive importance of these 14 genera, with *Roseburia* emerging as the most important in distinguishing between Female_Health and Female_HUA. *Roseburia* was significantly enriched in the Female_Health group but showed no significant difference between Male_HUA and Male_Health in either the discovery or validation cohorts (Figure 4C). Therefore, *Roseburia* may serve as a key sex‐specific protective genus that helps females resist HUA.

### *Roseburia* alleviated HUA in females by upregulating the production of glycine from xanthine

**FIGURE 5:**
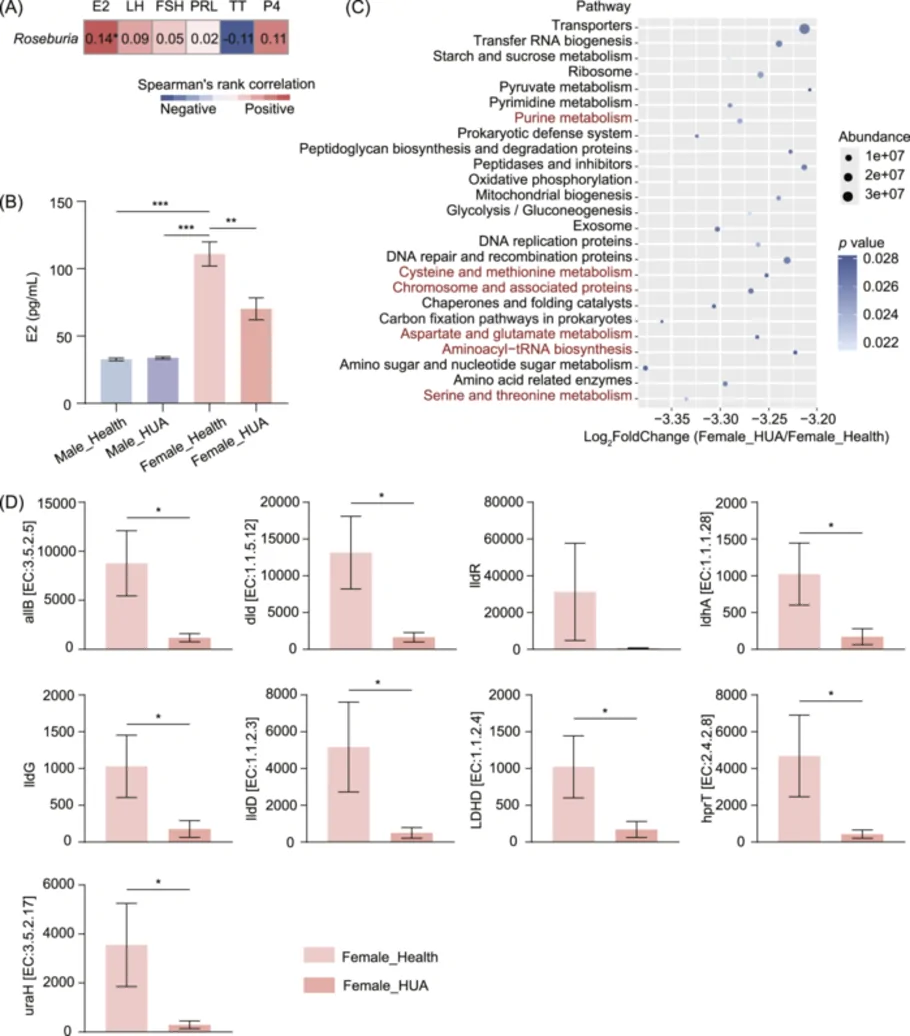
Functional differences in the gut microbiome between diseased and healthy Females. (A) Heatmap represented the correlation between *Roseburia* and sex hormones in females (E2: estradiol; LH: luteinizing hormone; FSH: follicle‐stimulating hormone; PRL: prolactin, TT: total testosterone; P4: Progesterone; Blue in the boxes indicates negative correlations, and red indicates positive correlations). (B) The bar graph represents the distribution of E2 in four groups of human samples. *p < 0.05, **p < 0.01, ***p < 0.001 analyzed by the Mann–Whitney *U* test. (C) The top 25 metabolic pathways were compared for females. Larger bubbles or darker colors indicated a higher abundance of that metabolic pathway within that group. (D) Distribution of key metabolites associated with UA metabolism in females (allB: allantoinase; dld: d‐lactate dehydrogenase; lldR: l‐lactate dehydrogenase operon regu; ldhA: d‐lactate dehydrogenase; lldG: l‐lactate dehydrogenase complex protein; lldD: l‐lactate dehydrogenase (cytochrome); LDHD: d‐lactate dehydrogenase; hprT: hypoxanthine phosphoribosyltransferase; uraH: 5‐hydroxyisourate hydrolase). *p < 0.05, **p < 0.01, ***p < 0.001 analyzed by the unpaired one‐tailed unpaired Student's *t*‐tests. UA, uric acid.

*Roseburia* had been identified as the marker genus that most effectively distinguished between premenopausal and postmenopausal females [25, 26]. In postmenopausal females, there were significant changes in hormone levels [27], which suggested that *Roseburia* reduced high UA in females, possibly through E2. Spearman's rank correlation analysis examined the relationship between *Roseburia* and sex hormones. The results showed that *Roseburia* significantly correlated with E2 (Figure 5A). E2 levels were significantly higher in the Female_Health group than in the other three groups, consistent with the abundance profile of *Roseburia* in the gut (Figure 5B). To further elucidate the specific mechanism of *Roseburia* in alleviating HUA in females, the key differential metabolic pathways between Female_Health and Female_HUA were screened (Figure 5C). The pathways of purine metabolism, cysteine, and methionine metabolism, as well as serine and threonine metabolism, were significantly enriched in Female_Health compared to the Female_HUA group, suggesting that these pathways may regulate UA production and excretion (Figure 5C). Lactate dehydrogenase and hypoxanthine phosphate acyltransferase, associated with UA excretion and metabolism, were more abundant in the Female_Health group than in the Female_HUA group (Figure 5D). Enriching metabolic pathways and enzymes associated with UA metabolism in the Female_Health group may explain why females were less likely to develop HUA.

### Mechanism network diagram of sex hormones regulating gut microbes and UA

**FIGURE 6:**
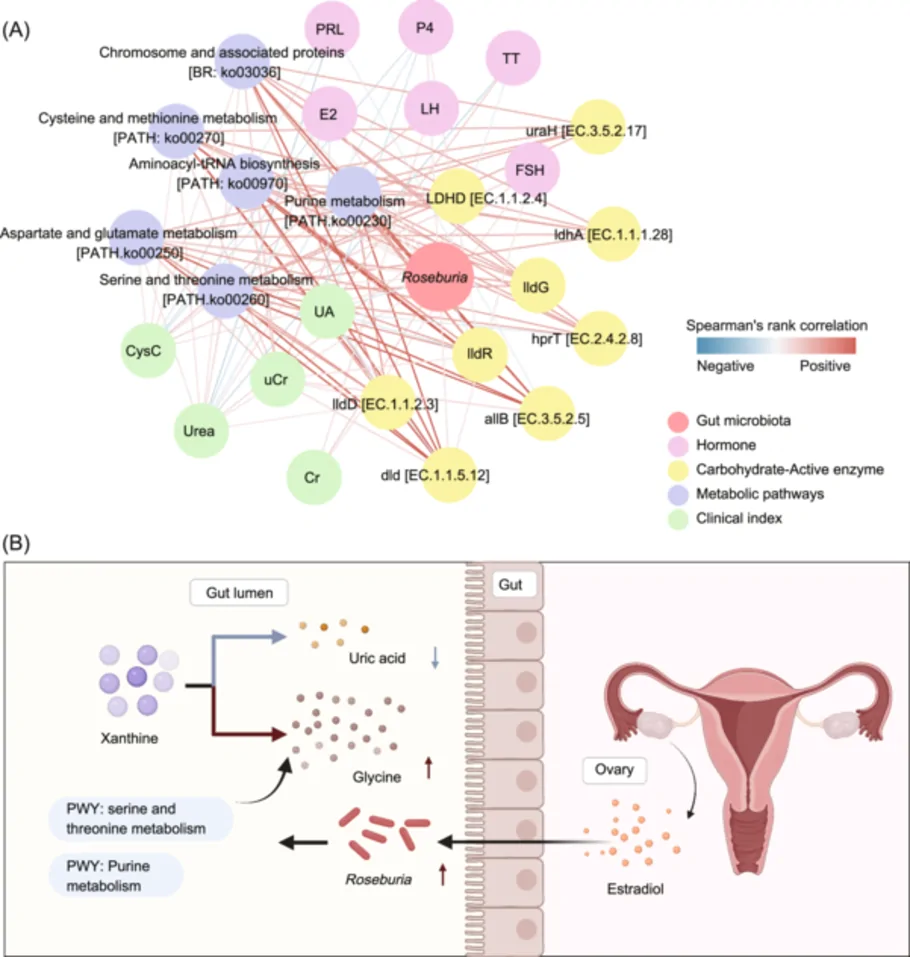
Mechanisms of *Roseburia* on HUA in females. (A) *Roseburia*, hormones, metabolic enzymes, metabolic pathways, and clinical indicators were represented by dots of different colors. The color of the edges (red for positive correlation and blue for negative correlation) was proportional to the strength of the correlation (E2: estradiol; LH: luteinizing hormone; FSH: follicle‐stimulating hormone; PRL: prolactin; TT: total testosterone; P4: Progesterone; allB: allantoinase; dld: d‐lactate dehydrogenase; lldR: l‐lactate dehydrogenase operon regu; ldhA: d‐lactate dehydrogenase; lldG: l‐lactate dehydrogenase complex protein; lldD: l‐lactate dehydrogenase (cytochrome); LDHD: d‐lactate dehydrogenase; hprT: hypoxanthine phosphoribosyltransferase; uraH: 5−hydroxyisourate hydrolase; Cr: creatinine; CysC: cystatin C; uCr: urinary creatinine). (B) Mechanisms by which E2 influenced *Roseburia* to relieve UA in females. HUA, hyperuricemia; UA, uric acid.

A network to visualize the correlations between *Roseburia*, clinical indicators, hormones, key metabolic pathways, and key metabolic enzymes were constructed in the female cohort using Spearman's rank correlation coefficients (Figure 6A). *Roseburia* was positively correlated with pathways involved in the conversion of xanthine to glycine, including chromosome‐associated proteins, purine metabolism, cysteine and methionine metabolism, aminoacyl‐tRNA biosynthesis, aspartate and glutamate metabolism, as well as serine and threonine metabolism. Among the key metabolic enzymes, *Roseburia* was positively correlated with d‐lactate dehydrogenase. Additionally, *Roseburia* exhibited positive correlations with E2 and progesterone (P4). Regarding kidney‐related indicators, *Roseburia* showed a negative correlation with UA and positive correlations with other kidney function indices, such as CysC, Cr, and urea (Figure 6A). Based on the above results, a potential mechanism was proposed: E2 may promote the enrichment of *Roseburia* by affecting the gut microbiota, and high levels of *Roseburia* may reduce the production of UA by enhancing the xanthine‐generating glycine metabolic pathway (Figure 6B). The abundance of *Roseburia* in healthy premenopausal females may serve as a protective mechanism, mitigating the risk of HUA by promoting metabolic processes that favor UA excretion and reducing its generation. E2 levels influence the enrichment of *Roseburia* in the female gut, highlighting the impact of gender‐specific microbes on UA regulation (Figure 6B).

## 3 DISCUSSION

In this large observational cohort, the relationship between HUA and gut microbiota was systematically investigated, and sex differences in the impact of this interaction were highlighted. By comparing the different genera between Male_Health and Female_Health cohorts, we identified genera that may represent those with potential protective significance for females without disease. By further comparing the Female_Health and the Female_HUA cohorts, we identified genera affected by HUA in females, thereby capturing disease‐specific microbial characteristics within the female population. Therefore, by integrating the differential genera identified through these two comparative approaches, we obtained a set of genera, including *Roseburia* and 28 other microbes, that meet the following criteria: (i) Gender‐specific differences, where these genera are enriched in Female_Health (potentially indicating gender‐specific protective roles) or depleted in Female_Health (potentially indicating gender‐specific harmful effects); (ii) Disease‐specific differences, where these genera are reduced in affected females (potentially indicating disease‐specific protective roles) or enriched in affected females (potentially indicating disease‐specific harmful effects). In conclusion, the differential genera we discovered are specifically enriched or depleted in Female_Health cohorts, possibly playing gender‐specific protective or promoting roles in the occurrence or progression of HUA. Among these, beneficial genera such as *Faecalibacterium*, *Roseburia*, and *Blautia* are more abundant in the Female_Health cohorts. These genera, capable of producing SCFAs, play a crucial role in UA metabolism and negatively correlate with UA levels [19, 21, 28].

After integrating the differential genera, we further collected 256 gut microbiome samples from three published HUA cohorts as a validation cohort to assess the universality of these genera in HUA patients. In the discovery and validation cohorts, genera such as *Roseburia* were significantly enriched only in the Female_Health cohort. Furthermore, a recently published study indicated that *Roseburia* negatively correlates with UA levels [24]. These results suggest that the gender‐ and disease‐specific enrichment of *Roseburia* indeed has universality. Subsequently, to identify key biomarkers contributing to the gender differences in HUA, we calculated the correlation between *Roseburia* and 13 other genera with UA levels. The results revealed that *Roseburia* and eight other genera were negatively correlated with UA levels. Notably, only *Roseburia* showed a significant negative correlation with UA levels. This finding underscores the significant association between *Roseburia* and HUA, indicating its potential protective role in the occurrence and development of HUA. Next, we performed random forest analysis using the discovery cohort of 623 individuals from our study and the validation cohort comprising 256 gut microbiome samples from previously published studies. This analysis further confirmed that *Roseburia* is the most critical genus that distinguishes between the Female_Health and the Female_HUA cohort. In conclusion, we identified *Roseburia* as a potential gender‐specific biomarker for female HUA by integrating differential and correlation analyses.

*Roseburia* was crucial in distinguishing healthy individuals from HUA patients, particularly in females. In addition to producing SCFAs, *Roseburia* stimulated Treg cell differentiation [28], enhanced host energy metabolism, exerted anti‐inflammatory effects, and protected the intestinal tract from pathogens and diseases [29]. Furthermore, *Roseburia* abundance was reduced in obese patients with HUA, hyperlipidemia, and hypertension [30]. Our findings suggest correlations between *Roseburia* and purine metabolism‐related pathways, particularly those that may promote the conversion of xanthine to glycine. *Roseburia* may influence host metabolic pathways and be associated with reduced UA levels.

Sex hormones, mainly E2, significantly impacted the gut microbiota, with E2 shown to regulate the growth and activity of a wide range of gut microbiota [31]. Differences in sex hormone levels between males and females emerged as a key factor contributing to the observed variations in gut microbiota [32]. Additionally, sex hormones play a crucial role in UA metabolism, acting as important regulatory signaling factors for both UA production and excretion [33]. As females age, particularly post‐menopause, the decline in E2 levels could lead to reduced UA clearance, thereby increasing the risk of HUA [34]. Sex hormones also affect the gut microbiota. Studies demonstrate that E2 promoted the attachment and colonization of *Botrytis cinerea* and inhibited colorectal cancer in female mice [35]. Higher E2 levels were associated with a greater abundance of *Bacteroidetes*, a lower abundance of *Firmicutes*, and increased microbial diversity [16]. Lower *Firmicutes*/*Bacteroidetes* ratios correlated with better intestinal homeostasis and overall health status [16]. Furthermore, gut microbiota dysbiosis due to a high‐fat diet in E2‐treated male mice altered the abundance of *Collinsella aerofaciens*, which was linked to colonic macrophage infiltration [36]. The study found a correlation between E2 levels, the female microbiome, and its metabolites and highlights the importance of maintaining appropriate E2 levels for effective control of UA levels.

While this study highlights the correlation between sex differences and HUA in gut microbiota, several limitations must be acknowledged. Firstly, as a cross‐sectional design, this study cannot provide direct evidence of causality. Secondly, although our sample size included 623 subjects, it may still be insufficient for thoroughly investigating the specific impacts of gender on HUA. Thirdly, the ethnic background of the participants in this study was relatively homogeneous, which limits the generalizability of our findings to diverse populations globally. Future research should adopt multi‐center, multi‐ethnic studies with larger cohorts to enhance the universality and reliability of the gender‐specific conclusions. Additionally, longitudinal studies or randomized controlled trials should be employed to understand better the relationships among gender, gut microbiota, and HUA. Although this study provides new insights into the relationship between gender differences and HUA, further investigations are necessary to lay a more solid theoretical foundation for clinical practice.

## 4 CONCLUSION

This study found that for HUA, there is a significant difference between male and female gut microbes and that females have more beneficial genera of gut microbes, which may be one of the reasons why females are less prone to HUA. In exploring the gender‐specific mitigating effects of gut microbes on HUA in females, we identified *Roseburia* as a key genus. Higher E2 levels positively correlated with *Roseburia*, allowing this bacterium to exert a more pronounced effect on reducing UA levels. In contrast, males lacked this E2‐mediated advantage, rendering them more susceptible to HUA. These findings not only enhanced our understanding of how sex hormones influence human health by modulating the gut microbiota but also provided a theoretical foundation for developing novel therapeutic strategies for HUA. Future research should focus on validating the potential of *Roseburia* as a probiotic and further exploring the mechanisms underlying the complex interactions between sex hormones, gut microbiota, and UA metabolism. This line of inquiry could pave the way for new approaches to combat HUA and related diseases, particularly in developing personalized medical solutions tailored to female health.

## 5 METHODS

### Recruitment of subjects

A cross‐sectional study was conducted on 623 subjects from Bo'ao Yiling Life Care Center, Hainan, China, to investigate the relationship between HUA and gut microbiota. All subjects in this study underwent a comprehensive physical examination. Fundamental indicators, such as height, weight, body mass index, and blood pressure were also measured. The study comprised male and premenopausal female subjects with HUA and healthy controls. HUA was defined as the level of serum UA exceeding 420 µmol/L for males and 360 µmol/L for premenopausal females, based on standard clinical guidelines [2]. Using these criteria, participants were divided into four groups: Male_Health (healthy males, *n* = 185), Male_HUA (males with HUA, *n* = 168), Female_Health (healthy females, *n* = 195), and Female_HUA (females with HUA, *n* = 75). To be included in the study, subjects had to meet the following criteria: be aged 40 or older, provide fecal, blood, and urine samples, refrain from taking medications or supplements (e.g., antibiotics or probiotics) for at least 3 weeks, and sign an informed consent form. These steps ensured the integrity of the study's data and participant compliance [24]. In addition, 256 population samples were collected as a validation cohort from three published cohorts associated with HUA, including healthy males, healthy females, males with HUA, and females with HUA [12, 18, 37].

### Clinical indicators and sex hormone level testing

Samples of peripheral venous blood were collected from all participants after 12 h of fasting. Serum biochemical indexes, liver function, kidney function, blood lipid levels, and hormone levels were measured using a Roche Cobas 602 automatic chemiluminescence analyzer. Specifically, the measured indicators included RBC count, HCT, mean corpuscular volume (MCV), IG, and Hb in the blood; kidney function was reflected via urea, UA, CysC, and Cr, as well as liver‐related ALP. Cardiovascular system‐related indicators such as LDL‐C, HDL‐C, ApoA1, apoB, and total cholesterol (TC) were also measured. In addition, six hormone levels were assessed, including E2, luteinizing hormone (LH), prolactin (PRL), follicle‐stimulating hormone (FSH), total testosterone (TT), and P4.

### Fecal sample collection and 16S rDNA sequencing

Each participant's fecal sample was collected in a sterile container and stored at −80°C [24]. Bacterial DNA was extracted from the samples using the bead‐beating method [38]. DNA quality was assessed by 0.8% agarose gel electrophoresis, and the OD 260/280 ratio was measured using a spectrophotometer [39]. The V4 region of the 16S rDNA gene was amplified using extracted DNA as a template and sequenced on the Illumina MiSeq platform [38].

### Identification of key microbial populations

End‐read merging of paired data was performed using VSEARCH (v2.28.1) [40]. Species annotation of DNA sequences was conducted by Kraken (v2.1.3), followed by Bracken (v2.9) to estimate the abundance of genera in the samples based on Kraken's classification results [41]. Based on this, a classification of the relative abundance in the gut microbiome was obtained. Using PICRUSt2 (v2.5.2), potential genes and metabolic pathways in gut microbiota were predicted by comparing the Kyoto Encyclopedia of Genes and Genomes (https://www.genome.jp/kegg/) based on 16S rDNA sequencing data [42].

### Statistical and bioinformatic analysis

Statistical analysis was performed using R (v4.3.3) software. The Mantel test was used to calculate the correlation between the gut microbiota and the two matrices of clinical indicators [43]. The Adonis test quantified the contribution of physical changes in subjects to the microbiome. Alpha diversity indices, such as the Shannon, Simpson, Chao1, and ACE index, were calculated to assess gut microbiota diversity. Beta diversity was assessed based on the Bray–Curtis distance and visualized using PCoA. The stacked column chart was drawn using the “ggplot” package. The Mantel test, Adonis test, alpha diversity, and Bray–Curtis distance were calculated using the “vegan,” “permute,” and “lattice” packages [44]. The differential genera were determined by the Mann–Whitney *U* test and were visualized using the “pheatmap” package in R software. The correlation was assessed using Spearman's rank correlation analysis. Random forests were employed to identify the key genera contributing to high female UA levels.

Differences in clinical indicators, genera, metabolic pathways, and hormone abundance were analyzed using the Mann–Whitney *U* test. Unpaired one‐tailed Student's *t*‐test were used to analyze differences in the abundance of metabolic enzymes. In the table presenting the basic characteristics of the study participants, data were presented as mean ± standard deviation (SD); *p*‐values were analyzed using the Mann‐Whitney U test. Both box and bar charts were constructed using GraphPad Prism (v8.0.2) software. The Spearman's rank correlation coefficient was calculated using the “psych” package and visualized by constructing network diagrams using Cytoscape (v3.10.2). Potential mechanism maps were constructed using BioRender (https://app.biorender.com) [44].

## AUTHOR CONTRIBUTIONS

**Zhihan Yang**: Formal analysis; investigation; data curation; writing—original draft; visualization. **Zeng Zhang**: Formal analysis; investigation; data curation; writing—original draft; visualization. **Mohan Chen**: Formal analysis; data curation. **Jin Hu**: Investigation; data curation; visualization. **Yingqi Qiu**: Investigation. **Shijia Lin**: Visualization. **Xiaolu Zhou**: Visualization. **Jiachao Zhang**: Formal analysis; writing—review and editing; supervision; funding acquisition. **Shuaiming Jiang**: Conceptualization; methodology; resources; writing—review and editing; supervision; project administration.

## CONFLICT OF INTEREST STATEMENT

The authors declare no conflicts of interest.

## 1 ETHICS STATEMENT

This study was a cross‐sectional clinical trial conducted in collaboration with Bo'ao Yiling Life Care Center from September 2023 to October 2023. The study was registered at the Chinese Clinical Trial Registry under the registration number ChiCTR2300075695 (https://www.chictr.org.cn/showproj.html?proj=202495) and approved by the Medical Ethics Committee of Bo'ao Yiling Life Care Center (No. IIT [2023] Clinical Ethics Review No. 001). Informed consent was required from all subjects before their participation.

## Supporting information

The online version contains a supplementary table available.

Table S1. Basic characteristics of the study participants.

## Data Availability

The data that support the findings of this study are openly available in Human feces Raw sequence reads at https://www.ncbi.nlm.nih.gov/bioproject/PRJNA1181621/, reference number PRJNA1181621. The metagenomic sequence data reported in this paper have been deposited into the NCBI genome database (https://www.ncbi.nlm.nih.gov/bioproject/PRJNA1181621/). The raw metabolomics identification and abundance data and more details on graph construction and reproducibility are available on GitHub (https://github.com/Yangzh987/Hyperuricemia-Gender-Differences-Project). Supplementary materials (tables, graphical abstract, slides, videos, Chinese translated version, and update materials) may be found in the online DOI or iMeta Science http://www.imeta.science/imetaomics/.
